# Lead (Pb) Toxicity; Physio-Biochemical Mechanisms, Grain Yield, Quality, and Pb Distribution Proportions in Scented Rice

**DOI:** 10.3389/fpls.2017.00259

**Published:** 2017-02-28

**Authors:** Umair Ashraf, Adam S. Kanu, Quanquan Deng, Zhaowen Mo, Shenggang Pan, Hua Tian, Xiangru Tang

**Affiliations:** ^1^Department of Crop Science and Technology, College of Agriculture, South China Agricultural UniversityGuangzhou, China; ^2^Scientific Observing and Experimental Station of Crop Cultivation in South China, Ministry of AgricultureGuangzhou, China

**Keywords:** antioxidant defense, grain yield, lead, oxidative stress, scented rice, quality characters

## Abstract

Lead (Pb) caused interruptions with normal plant metabolism, crop yield losses and quality issues are of great concern. This study assessed the physio-biochemical responses, yield and grain quality traits and Pb distribution proportions in three different fragrant rice cultivars i.e., Meixiangzhan-2, Xinagyaxiangzhan and Basmati-385. Plants were exposed to 400, 800, and 1,200 ppm of Pb while pots without Pb were taken as control (0 ppm). Our results showed that Pb toxicity significantly (*P* < 0.05) reduced photosynthetic pigments (chlorophyll contents and carotenoids) and inducted oxidative stress with increased production of hydrogen peroxide (H_2_O_2_), malanodialdehyde (MDA) and leaves leachates; while such effects were more apparent in Xinagyaxiangzhan than other two rice cultivars. Pb stress differentially affected the production protein, proline and soluble sugars; however the production rates were higher at heading stage (HS) than maturity stage (MS). Furthermore, Pb stress altered superoxide dismutase (SOD), peroxidases (POD), catalases (CAT) and ascorbate peroxidases (APX) activities and glutathione (GSH) and oxidized glutathione (GSSG) production in all rice cultivars at both HS and MS. All Pb levels reduced the yield and yield components of all rice cultivars; nonetheless such reductions were observed highest in Xinagyaxiangzhan (69.12%) than Meixiangzhan-2 (58.05%) and Basmati-385 (46.27%) and resulted in grain quality deterioration. Significant and positive correlations among rice yields with productive tillers/pot and grains per panicle while negative with sterility percentage were also observed. In addition, all rice cultivars readily taken up the Pb contents from soil to roots and transported upward in different proportions with maximum in roots followed by stemss, leaves, ears and grains. Higher proportions of Pb contents in above ground plant parts in Xinagyaxiangzhan possibly lead to maximum losses in this cultivar than other two cultivars; while less damage in Basmati-385 might be related to strong anti-oxidative defense system and lower proportions of Pb contents in its aerial parts.

## Introduction

Recent rates of soil contamination with different heavy metals (the non-essential elements for plants) and their entrance in to the agro-ecosystems and transference to human beings through food chain is an alarming situation over the globe (Abrahams, 2002; Anjum et al., 2016a,b). In terrestrial ecosystems, soil act as the main source of heavy metal transference to agricultural products. These metals enter in to the plant systems from soil or from external atmosphere surrounded by the plant and would have serious consequences in crop productivity and grain qualities.

Among different heavy metals, lead (Pb), is the second most harmful pollutant after arsenic and recently listed as “the chemical of great concern” according to the new European REACH regulations (Pourrut et al., 2011). It severely affects normal plant metabolism, morph-physiological features and crop growth and productivity (Sharma and Dubey, 2005; Ashraf et al., 2015). It often leads to diminished growth, deformation of cellular structures, ion homeostasis, reductions in chlorophyll biosynthesis, hormonal imbalance and induce over-production of reactive oxygen species (ROS) in plants (Shahid et al., 2011; Kumar et al., 2012). Pb, being non-redox metal, cause ROS production that led to oxidative stress within plant cells (Singh et al., 2010). Once produced, these ROS readily attacks to biological structures and biomolecules and results in metabolic dysfunction (Clemens, 2006).

Plants normally have three mechanisms of Pb-tolerance i.e., (a) passive mechanisms (plant develops different types of physical barriers against Pb uptake), (b) inducible mechanisms (metal detoxification and its excretion to extra-cellular spaces), and (c) activation of anti-oxidative defense system (which includes both enzymatic and non-enzymatic anti-oxidants) to scavenge ROS (Pourrut et al., 2011; Ashraf et al., 2015). Anti-oxidants, both enzymatic such as superoxide dismutase (SOD), peroxidase (POD), catalase (CAT), and ascorbate peroxidase (APX), and non-enzymatic such as reduced glutathione (GSH) and oxidized glutathione (GSSG) are involved in direct and/or indirect detoxification of ROS in plants (Mishra and Choudhary, 1998; Mittler, 2002). Except anti-oxidants, plants also accumulate various types of organic compounds or osmolytes proline and soluble sugars to shield essential cellular structures and to maintain cell osmotic potential (Chatterjee et al., 2004; Ali et al., 2014).

Scented rice is the most popular rice type among farmers and consumers over the globe due to its special aroma (Singh et al., 2000). Increasing demands and opportunities to get premium prices in world markets, yield maintenance and quality assurance are the main factors that would lead it to the international markets to meet consumer requirements. However, soil contaminated with toxic metals, especially Pb, would thus become the main hindrance in its way as it readily absorbed by the roots and transports to the edible plant parts which often lead to rice quality deterioration. Rice milling quality can be assessed by the rate of brown rice, milled rice, and head rice rates as well as milling recovery percentages which directly related to the market value while rice chalkiness percentage, chalkiness degree and grain protein and amylose contents are the appearance and cooking qualities of rice grains (Fahad et al., 2016a) that would greatly affected by Pb contaminated soils. Hence Pb effects on rice physiology and its mimicking effects on yield and quality as well as grain Pb concentrations are important to study. Although many previous studies reported the effects of Pb on rice growth and physio-biochemical responses but to our knowledge, a very few work has been done on its effects on yield and quality characters along with physio-biochemical responses especially in aromatic rice. Present study was planned with the objectives to (a) assess the Pb-induced changes in plant physio-biochemical mechanisms including anti-oxidant activities, (b) investigate the changes in yield and quality traits of aromatic rice, and to (c) estimate percentage proportions of accumulated Pb in different plant parts of rice.

## Materials and methods

### Experimental site, soil, and conditions

The experiment was carried out in open air conditions under rain-protected wire house located at Experimental Research Farm, College of Agriculture, South China Agricultural University, Guangzhou, China (23°09′ N, 113°22′ E and 11 m above the sea level) during April to July 2015 as early season rice in South China. During growing season, the mean monthly day and night temperatures lies between 23–29°C and 22–28°C, respectively with average humidity between 77 and 89%.

One month prior to nursery transplanting, the pots were filled with air-dried soil collected from uncontaminated paddy field with 10 kg soil in each pot (25 cm in height and 32 cm in diameter) containing total N 1.190 g kg^−1^, total P 0.952 g kg^−1^, total K 18.30 g kg^−1^, available N 90.38 mg kg^−1^, available P 9.45 mg kg^−1^, available K 130.29 mg kg^−1^, organic matter 21.83 g kg^−1^, soil pH 5.63, and 51.12 ppm initial soil Pb contents.

### Treatment application, nursery transplantation, and crop husbandry

After pot filling, soil within pots was supplemented with Pb by using Pb(NO_3_)_2_ in solution form. The required Pb concentrations i.e., 400, 800, and 1,200 ppm were prepared by dissolving the Pb(NO_3_)_2_ salt in deionized water, applied in respective pots and thoroughly mixed at the same time. The pots without any addition of external Pb were regarded as control (0 ppm). The pots were kept in submerged conditions by maintain a water layer above soil surface to create fully anaerobic and puddle like conditions within the pots.

Seeds of three aromatic rice cultivars i.e., “Meixiangzhan-2” (Lemont × Fengaozhan, indica type of aromatic rice cultivar with 145–147 days to maturity), “Xiangyaxiangzhan” (Xiangsimiao126 × Xiangyaruanzhan, indica type of aromatic rice cultivar with 112–114 days to maturity), and “Basmati-385” (early maturing, intermediate tall with very good cooking quality and aroma with 145–150 days to maturity) were collected from the College of Agriculture, South China Agricultural University, Guangzhou, China and soaked in tap water in wooden box at 25–30°C for 48 h to complete germination process. Healthy and germinated seeds were subsequently sown on soil containing plastic trays and placed on leveled paddy field and covered with a plastic sheet in early March. One month old seedlings were then transplanted to the pots with 3–4 seedlings per hill and 5 hills per pot (four seedlings aside and one in the middle of the pot). Till 1 week after transplanting, the damaged, broken and died seedlings were replaced with the healthy seedlings to get a good stand establishment. All pots were applied with 2.25 g N (Urea 46%), 3.33 g P (super phosphate 12% P_2_O_5_), and 1.35 g K (potassium chloride 60% K_2_O) with 70% as starter dose and 30% at tillering stage. The plants were regularly monitored and a water layer of 2–3 cm was maintained throughout the growth period with tap water.

### Sampling and observations

For physio-biochemical analyses and Pb determination, plants were sampled at heading stage (HS) and maturity stage (MS) and stored at −80°C (for biochemical assays) while yield and quality attribute were recorded at MS.

### Photosynthetic pigments and biochemical assays

To determine photosynthetic pigments, fresh leave samples (0.2 g) at HS and MS were extracted with 15 ml of 95% ethanol and the contents were estimated according to Arnon (1949). Hydrogen peroxide (H2O2) contents were estimated according to Velikova et al. (2000). The malondialdehyde (MDA) contents were determined by following the methods of Hodges et al. (1999) with the following formula: MDA content = {6.45(ΔOD_532−600_) − (0.56OD_450_)}. To estimate leachates percentage, the fresh leaf samples were thoroughly washed with deionized water (Milli-Q), cut in to small pieces. Leaf discs (0.3 g) were then placed in 10 ml deionized water and incubated at 25°C for 6 h and the electrical conductivity (EC) (EC_1_) was recorded with an EC meter (SX-650, Sansin, China). To record the second EC (EC_2_), the samples were then incubated at 90°C for 2 h and then cool down to 25°C. The leachates of the leaves samples were calculated as: leachates (%) = EC_1_/EC_2_ ×100) (Valentovic et al., 2006). Protein contents of fresh leaves samples were estimated according to Bradford (1976) at HS and MS using G-250. The Proline contents in fresh leaves samples (0.2 g) were determined according to Bates et al. (1973) by using ninhydrin at both HS and MS. For soluble sugars determination at HS and MS, methods of Zhang et al. (2009) were followed.

Fresh leaves samples (0.3 g) were crushed in liquid-N_2_ and homogenized in 6 ml of 50 ml sodium phosphate buffer (pH 7.8) with mortar and pestle in an ice bath and homogenate were centrifuged at 10,000 g for 20 min at 4°C and the aliquot of the supernatant was used to record the enzymatic activities.

Activities of superoxide dismutase (SOD, EC 1.15.1.1) were assayed according to Zhang et al. (2008) by following the inhibition of photochemical reduction due to nitro blue tetrazolium (NBT). SOD activity per unit was defined as the amount of enzyme required to inhibit NBT photochemical reduction to 50% as an activity unit (U). Peroxidase (POD, EC 1.11.1.7) activity was measured by using guaiacol method with minor modifications. The reactions mixture contained 1 ml of sodium phosphate buffer (pH 7.8), 0.2% of 0.95 ml guaiacol, 1 ml of 0.3% H_2_O_2_, and 0.05 ml aliquot of enzyme extract. The absorbance was read at 470 nm (Zhou and Leul, 1999). One unit of POD activity was the amount of enzyme that caused the decomposition of 1 μg substrate at 470 nm. Catalase activity (CAT, EC 1.11.1.6) was assayed according to the procedures of Aebi (1984) whereas one unit of enzyme activity (U) was the decomposition of 1 M H_2_O_2_ at A_240_ within 1 min in 1 g of fresh leaves samples. Ascorbate peroxidase (APX, EC 1.11.1.11) activity was estimated by using “APX determination kit” purchased from Nanjing Jiancheng Bioengineering Institute, China. The methods devised by the manufacturer were followed carefully and the absorbance was read at 290 nm. Reduced glutathione (GSH) and oxidized glutathione (GSSG) were also estimated by using the readymade build kits (A006-1 for GSH and A061-2 for GSSG), provided by Nanjing Jiancheng Bioengineering Institute, China (http://www.njjcbio.com). The instructions were strictly followed and the absorbance was read at 420 and 412 nm, respectively. Total glutathione was the addition of both GSH and GSSG (GSH+GSSG) contents.

### Grain yield and related attributes

To determine yield and related attributes, plants were harvested at MS and threshed manually for all rice cultivars and the grains were sun-dried till the grain moisture contents were lowered to 12–14%. Total tillers were calculated in each pot at tillering stage, while at reproductive stage; tillers containing panicles in each pot were counted to represent the productive tillers per pot. Spikelets were separated from the panicles of all rice cultivars and spikelets per panicle were calculated in each treatment. Number of partially filled and totally unfilled/empty grains on each panicle was counted and sterility percentage was calculated. Six random samples of 1,000-grains were taken from filled seed lot of all rice cultivars and weighed to get 1,000-grain weight and expressed in grams (g) while the total paddy weight from each pot was represented as grain yield pot^−1^. The harvest index of all rice cultivars was calculated as: 100 × (grain yield/plant dry biomass).

### Grain quality characters

The harvested grains were taken from stored seed lot to determine rice quality attributes. A rice huller (Jingsu, China) was used to get brown rice and brown rice rate (BRR) was calculated as: (brown rice weight/paddy weight) × 100. The brown rice was milled with a Jingmi testing rice miller (Zhejiang, China) and milling recovery was recorded as: (milled rice weight/original weight) × 100. Milling degree was calculated as: milled rice weight/brown rice weight) × 100. To calculate head rice rate, whole milled grains were separated from 100 grains and head rice rate was calculated on percentage basis. Grain chalkiness percentage and chalkiness degree were assessed with the help of an SDE-A light box (Guangzhou, China). Grain chalkiness is the grains having opaque spots either on the dorsal and/or in center of the grain. Grain moisture, amylose, protein contents and grain alkali were determined by using an Infratec 1241 (FOSS-TECATOR) grain analyser.

### Pb-determination in different plant parts

The sampled plants were separated into roots, stems, leaves, ears (at PH) and grains (at MS) stage, respectively of each rice cultivar and placed in oven at 80°C for drying. The oven dried ground samples (0.2 g) of each part were digested with di-acidic mixture of HNO_3_:HClO_4_ with a proportion of 4:1 *v/v*, and resultant solutions were diluted to 50 ml and then filtered by using Whatman filter paper no. 1. The Pb-concentrations in respective samples were estimated by using Atomic Absorption Spectrophotometer (AA6300C, Shimadzu, Japan) and the proportions Pb contents in different plant parts were calculated on percentage basis.

### Experimental design and statistical analyses

Pots were arranged in randomized complete block design (RCBD), having enough space among them to avoid shading, with 10 pots per treatment. The data were analyzed by using a statistical software “Statistix 8” (Analytical software, Tallahassee, Florida, USA) whilst difference amongst treatments were separated using least significant difference (LSD) test at *P* < 0.05. Correlation analyses among grain yield vs. other yield related attributes were computed and represented by using SigmaPlot 9.0 (Systat Software Inc., San Jose, CA, USA).

## Results

### Chlorophyll contents and carotenoids

Pb stress inhibited photosynthetic pigments, i.e., Chl a, Chl b, total chlorophyll contents (Chl a+b) and carotenoids significantly (*P* < 0.05) and the inhibition rates were increased with an increase in Pb-toxicity with highest at 1,200 ppm in all rice cultivar. For example, at heading stage (HS), the values for percentage reduction in Chl a, Chl b, and total chlorophyll contents (Chl a+b) were recorded as 54.80, 54.68, and 54.75% in Meixiangzhan-2, 54.41, 56.73, and 87.76% in Xiangyaxianzhan and 35.66, 37.07, and 36.21% in Basmati-385, respectively as compared with control. Similarly, at maturity stage (MS), the percentage reduction for Chl a, Chl b, and total chlorophyll contents (Chl a+b) were remained as 51.69, 39.63, and 34.56% for Meixiangzhan-2, 52.43, 59.69, and 55.46% for Xiangyaxianzhan, and 41.86, 50.33, and 45.11% for Basmati-385, when compared with control. Moreover, the ratio of Chl a/b were remained higher at higher doses of Pb-toxicity while chlorophyll contents were affected more severely due to Pb toxicity than carotenoids in all rice cultivars. Overall, the chlorophyll contents and carotenoids were recorded higher at HS as compare to MS while the magnitude of Pb stress on photosynthetic pigments across cultivars was recorded as: Xiangyaxianzhan > Meixiangzhan-2 > Basmati-385 (Table 1).

**Table 1:** **Effect of different Pb concentrations on photosynthetic pigments of three scented rice cultivars at heading and maturity stages**.

**Rice cultivars**	**Pb (ppm)**	**HS**					**MS**				
		**Chl a**	**Chl b**	**Chl a+b**	**Carotenoids**	**Chl a/b**	**Chl a**	**Chl b**	**Chl a+b**	**Carotenoids**	**Chl a/b**
		**mg g**^**−1**^ **FW**					**mg g**^**−1**^ **FW**				
Meixiangzhan-2	0	3.22 ± 0.32a	2.12 ± 0.46a	5.34 ± 0.74a	1.27 ± 0.21a	1.65 ± 0.33a	2.80 ± 0.46a	1.87 ± 0.23a	4.67 ± 0.58a	1.01 ± 0.07a	1.52 ± 0.23a
	400	1.60 ± 0.19b	1.03 ± 0.12b	2.63 ± 0.31b	1.15 ± 0.16a	1.55 ± 0.01a	2.21 ± 0.36ab	1.20 ± 0.17b	3.40 ± 0.36b	0.70 ± 0.12ab	1.96 ± 0.53a
	800	1.46 ± 0.02b	0.96 ± 0.01b	2.42 ± 0.03b	1.04 ± 0.34a	1.51 ± 0.02a	1.35 ± 0.11bc	1.13 ± 0.18b	2.48 ± 0.11*b*c	0.66 ± 0.15b	1.30 ± 0.35a
	1,200	1.15 ± 0.29b	0.56 ± 0.08b	1.72 ± 0.27b	0.59 ± 0.12a	2.18 ± 0.74a	1.12 ± 0.07c	0.57 ± 0.18b	1.69 ± 0.13c	0.55 ± 0.02b	2.63 ± 1.09a
Means		1.86	1.17	3.03	1.01	1.72	1.87	1.19	3.06	0.73	1.85
LSD _0.05_		0.78	0.79	1.38	0.73	1.32	0.98	0.63	1.15	0.33	2.09
Xiangyaxianzhan	0	2.21 ± 0.42a	1.52 ± 0.73a	3.73 ± 0.06a	1.83 ± 0.10a	1.61 ± 0.17a	2.02 ± 0.08a	1.45 ± 0.21a	3.47 ± 0.28a	0.96 ± 0.11a	1.43 ± 0.14a
	400	1.19 ± 0.02b	0.79 ± 0.02a	1.97 ± 0.18b	1.37 ± 0.34ab	1.47 ± 0.01a	1.11 ± 0.21b	0.76 ± 0.17b	1.87 ± 0.10b	0.65 ± 0.17ab	1.76 ± 0.72a
	800	0.97 ± 0.11b	0.66 ± 0.07a	1.62 ± 0.03b	1.02 ± 0.26b	1.51 ± 0.01a	0.96 ± 0.14bc	0.59 ± 0.05bc	1.55 ± 0.19b	0.39 ± 0.03b	1.63 ± 0.13a
	1,200	0.65 ± 0.06b	0.40 ± 0.02a	1.05 ± 0.83b	0.75 ± 0.04b	2.31 ± 1.20a	0.59 ± 0.05c	0.28 ± 0.07c	0.87 ± 0.10c	0.38 ± 0.09b	2.24 ± 0.41a
Means		1.26	0.84	2.09	1.24	1.73	1.17	0.77	1.94	0.60	1.77
LSD _0.05_		0.71	1.19	1.39	0.72	1.98	0.44	0.46	0.60	0.37	1.39
Basmati-385	0	4.47 ± 0.16a	2.85 ± 0.12a	7.32 ± 0.24a	1.82 ± 0.13a	1.57 ± 0.06a	3.86 ± 0.05a	2.40 ± 0.11a	6.27 ± 0.06a	1.46 ± 0.29a	1.61 ± 0.09a
	400	3.62 ± 0.08b	2.57 ± 0.15a	6.19 ± 0.21b	1.39 ± 0.21ab	1.41 ± 0.06a	3.33 ± 0.35a	2.31 ± 0.34a	5.64 ± 0.51a	1.26 ± 0.20ab	1.51 ± 0.30a
	800	2.87 ± 0.27bc	1.80 ± 0.28b	4.67 ± 0.06c	1.20 ± 0.08b	1.75 ± 0.49a	2.25 ± 0.15b	1.19 ± 0.09b	3.44 ± 0.16b	1.15 ± 0.12ab	1.91 ± 0.20a
	1,200	2.12 ± 0.34c	1.18 ± 0.07c	3.30 ± 0.35d	0.97 ± 0.18b	1.81 ± 0.30a	2.06 ± 0.06b	1.10 ± 0.04b	3.16 ± 0.06a	0.82 ± 0.09b	1.89 ± 0.11a
Means		3.27	2.10	5.37	1.35	1.64	2.88	1.75	4.63	1.17	1.73
LSD _0.05_		0.76	0.56	0.78	0.52	0.96	0.63	0.61	0.89	0.61	0.63

*Values are the means of three independent determinations ± SE. Values with different lower case letters differ significantly at P < 0.05 according to least significant difference test. HS, Heading stage; MS, Maturity stage*.

### H_2_O_2_, MDA contents, leaf leachates, and osmo-regulation

The degree of H_2_O_2_ production, lipid peroxidation (in terms of MDA production) and leaf leachates (an indicator of membrane damage) is associated with the Pb-toxicity level. Significant increase (*P* < 0.05) in MDA, H_2_O_2_ contents and leachates were recorded with an increase in soil Pb-concentrations in all three rice cultivars at both HS and MS. For Meixiangzhan-2, the values of percentage increase were 30.79–126.53% and 17.80–79.45%, 34.34–157.34% and 1.75–46.14% and 95.60–116.27% and 62.96–133.07% in H_2_O_2_, MDA and leaf leachates at HS and MS, respectively. Similarly, the values of percentage increase in H_2_O_2_ were in the range of 99.16–156.01% and 33.73–68.04% (in Xiangyaxianzhan), 16.43–24.71% and 7.65–20.05% (in Basmati-385), in MDA were in the range of 12.52–151.48% and 53.97–104.88% (in Xiangyaxianzhan) and 76.75–108.43% and 11.83–25.27% (in Basmati-385), and in leaf leachates were recorded in the range of 61.18–112.46% and 77.13–130.19 (in Xiangyaxianzhan) and 66.06–79.21% and 97.46–100.50% (in Basmati-385), at HS and MS, respectively. In general, the degree of oxidative stress was highest in Xiangyaxianzhan followed by Meixiangzhan-2 and Basmati-385 while the values of percentage increase for MDA and H_2_O_2_ contents and leaf leachates were higher at MS than HS (Table 2).

**Table 2:** **Effect of different Pb concentrations on oxidative stress indicators, protein and osmolyte accumulation of three scented rice cultivars at heading and maturity stages**.

**Rice cultivars**		**HS**			**MS**			**HS**			**MS**		
	**Pb (ppm)**	**H_2_O_2_ (μmol g^−1^ FW)**	**MDA (μmol g^−1^ FW)**	**Leachates (%)**	**H_2_O_2_ (μmol g^−1^ FW)**	**MDA (μmol g^−1^ FW)**	**Leachates (%)**	**Protein (μg g^−1^ FW)**	**Proline (μg g^−1^ FW)**	**Soluble Sugars (mg g^−1^ FW)**	**Protein (μg g^−1^ FW)**	**Proline (μg g^−1^ FW)**	**Soluble Sugars (mg g^−1^ FW)**
Meixiangzhan-2	0	48.83 ± 3.80b	8.92 ± 0.07c	38.11 ± 0.41d	91.38 ± 3.46c	5.96 ± 0.24b	39.00 ± 0.22d	84.25 ± 4.04a	19.62 ± 2.84b	9.38 ± 0.60a	23.51 ± 3.17b	20.60 ± 3.84c	9.86 ± 0.59a
	400	63.86 ± 2.61b	11.98 ± 0.42b	74.54 ± 0.52c	107.65 ± 2.05bc	6.07 ± 0.22ab	63.56 ± 0.28c	88.01 ± 6.54a	16.30 ± 0.52b	9.84 ± 0.58a	39.74 ± 0.23a	24.12 ± 2.22bc	9.10 ± 0.53a
	800	90.66 ± 1.82a	10.44 ± 0.38bc	79.65 ± 0.46b	121.25 ± 5.70b	6.75 ± 0.22ab	79.54 ± 0.15b	109.94 ± 8.52a	21.77 ± 1.47b	10.13 ± 0.59a	39.27 ± 1.88a	39.35 ± 1.22a	9.56 ± 0.56a
	1,200	110.66 ± 7.61a	22.95 ± 1.16a	82.42 ± 0.26a	163.99 ± 7.36a	8.71 ± 1.59a	90.91 ± 1.04a	101.54 ± 12.07a	42.87 ± 2.06a	10.57 ± 0.55a	22.77 ± 7.70b	29.39 ± 0.68b	10.30 ± 0.55a
Means		78.50	13.57	68.68	121.07	6.87	68.25	95.94	25.14	9.98	31.32	28.37	9.71
LSD _0.05_		26.22	2.10	1.39	16.54	2.68	1.81	27.17	6.26	1.97	13.92	7.58	2.09
Xiangyaxianzhan	0	39.55 ± 4.90c	10.21 ± 0.24b	40.29 ± 1.54d	103.08 ± 3.21c	9.10 ± 0.85c	40.76 ± 1.23c	41.50 ± 2.00ab	12.98 ± 1.76b	9.46 ± 0.57a	39.00 ± 2.82a	21.97 ± 1.28b	9.19 ± 0.60a
	400	78.76 ± 1.18b	11.49 ± 1.26b	64.95 ± 0.08c	137.85 ± 6.42b	14.01 ± 0.66b	72.21 ± 0.17b	45.01 ± 0.31ab	31.54 ± 3.14a	9.92 ± 0.58a	36.76 ± 4.49ab	23.33 ± 1.98b	9.64 ± 0.55a
	800	80.20 ± 1.14b	11.51 ± 0.84b	70.68 ± 0.75b	168.83 ± 5.56a	16.94 ± 1.00ab	77.06 ± 5.75b	48.39 ± 2.10a	35.44 ± 4.18a	9.85 ± 0.60a	34.47 ± 2.05ab	24.51 ± 0.70b	10.17 ± 0.58a
	1,200	101.25 ± 0.26a	25.67 ± 2.44a	85.61 ± 0.14a	173.21 ± 5.37a	18.64 ± 1.30a	93.84 ± 0.86a	39.87 ± 3.67b	37.45 ± 5.62a	10.44 ± 0.55a	25.13 ± 6.02b	33.69 ± 0.52a	9.58 ± 0.60a
Means		74.94	14.72	65.38	145.74	14.67	70.97	43.69	29.35	9.92	33.84	25.88	9.65
LSD _0.05_		8.44	4.70	2.81	17.20	3.19	9.70	7.65	12.84	1.96	13.50	4.10	2.09
Basmati-385	0	95.50 ± 5.69*a*b	6.44 ± 0.95b	40.09 ± 0.49c	96.61 ± 6.67b	6.37 ± 1.27a	42.47 ± 3.39b	36.02 ± 0.53b	14.15 ± 1.79b	9.53 ± 0.56a	27.91 ± 7.30a	13.50 ± 2.69b	9.26 ± 0.54a
	400	92.88 ± 4.74b	11.39 ± 1.20a	66.57 ± 1.75b	103.99 ± 2.44ab	7.12 ± 1.35a	83.87 ± 0.32a	38.78 ± 2.20b	18.45 ± 1.86b	10.14 ± 0.60a	33.32 ± 2.20a	13.37 ± 1.60b	9.87 ± 0.57a
	800	111.18 ± 1.90a	11.40 ± 0.61a	69.18 ± 0.32ab	107.39 ± 3.14ab	7.20 ± 0.47a	86.76 ± 5.14a	45.01 ± 9.79b	20.01 ± 1.79b	10.13 ± 0.60a	40.69 ± 1.71a	15.33 ± 0.59b	9.86 ± 0.58a
	1,200	109.09 ± 6.60ab	13.43 ± 0.85a	71.84 ± 1.67a	115.97 ± 4.31a	7.98 ± 0.13a	85.16 ± 2.26a	76.06 ± 2.42a	31.15 ± 2.89a	10.45 ± 0.59a	39.40 ± 1.76a	29.00 ± 2.58a	10.17 ± 0.5a
Means		102.16	10.67	61.92	105.99	7.17	74.57	48.97	20.94	10.06	35.33	17.82	9.79
LSD _0.05_		16.48	3.03	4.07	14.48	3.12	10.70	16.85	6.96	1.92	13.06	6.68	2.09

*Values are the means of three independent determinations ± SE. Values with different lower case letters differ significantly at P < 0.05 according to least significant difference test. HS, Heading stage; MS, Maturity stage*.

Pb toxicity regulated the production protein, proline and soluble sugars in all rice cultivars at both HS and MS; however their concentrations were remained lower at MS than HS. Compared with control, the protein and proline contents of all rice cultivars (except protein contents in Meixiangzhan-2 at HS and in Basmati-385 at MS) were significantly affected (*P* < 0.05) by different Pb treatments, however, the contents of soluble sugars were remained statistically similar (*P* > 0.05) at both sampling stages. On average, at HS, Meixiangzhan-2 accumulated highest protein and proline contents while Basmati-385 accumulated higher soluble sugars at this stage whilst at MS, higher protein and soluble sugars were recorded in Basmati-385 (Table 2).

### SOD, POD, CAT, and APX activities

All Pb application levels affect variably to the enzymatic anti-oxidants in terms of SOD, POD, CAT, and APX activities in all rice cultivars at both HS and MS. At HS, the activity of SOD increased up to 25.53 and 32.66% at 400 ppm for Meixiangzhan-2 and Xiangyaxianzhan, respectively and then started to decline till 1,200 ppm; however for Basmati-385, the SOD activities were increased up to 49.41% till 800 ppm and then declined. Even though Pb toxicity changed SOD activities in Basmati-385 but the increase was not reached up to significance level (*P* > 0.05). The maximum POD activity was recorded at 400 ppm (63.46%) for Meixiangzhan-2, at 800 ppm (11.74%), and at 1,200 ppm (59.72%) for Basmati-385, compared with control. Similarly, the activities of CAT and APX were remained highest at 400 ppm (16.32 and 20.59%) for Meixiangzhan-2, at 800 and 1,200 ppm (4.62 and 134.78%) for Xiangyaxianzhan, and 5.38 and 58.33% for Basmati-385 at the same level of Pb as compared with control. At MS, the SOD activity decreased for Meixiangzhan-2, statistically similar (*P* > 0.05) for Xiangyaxianzhan and increased for Basmati-385 at all levels of Pb then control. The POD and CAT were recorded highest at 400 and 800 ppm (49.86 and 90.41%) and (2.04 and 57.29%) for Meixiangzhan-2 and Xiangyaxianzhan, respectively as well as at 1,200 ppm (18.53 and 44.76%) for Basmati-385. Similarly at HS, the activities of APX were also found statistically similar (*P* > 0.05) for all rice cultivars under in all Pb-treatments including control. Overall, the enzymatic activities were remained comparatively higher at HS than MS in all rice cultivars (Table 3).

**Table 3:** **Effect of different Pb concentrations on enzymatic anti-oxidants of three scented rice cultivars at heading and maturity stages**.

**Rice cultivars**	**Pb**	**HS**				**MS**			
		**SOD**	**POD**	**CAT**	**APX**	**SOD**	**POD**	**CAT**	**APX**
		**U min**^**−1**^ **g**^**−1**^ **FW**				**U min**^**−1**^ **g**^**−1**^ **FW**			
Meixiangzhan-2	0	405.94 ± 15.46ab	77.73 ± 0.27b	55.53 ± 2.34a	0.25 ± 0.06a	335.16 ± 39.66a	70.20 ± 0.76b	9.73 ± 0.77c	0.30 ± 0.05a
	400	509.59 ± 47.18a	127.07 ± 4.25a	64.60 ± 6.13a	0.30 ± 0.08a	205.94 ± 18.82b	105.20 ± 2.64a	14.51 ± 0.44b	0.38 ± 0.12a
	800	345.66 ± 23.05bc	81.93 ± 3.82b	28.07 ± 0.87b	0.15 ± 0.01a	211.42 ± 17.26b	51.40 ± 3.62c	18.54 ± 0.47a	0.51 ± 0.06a
	1,200	242.47 ± 44.82c	99.00 ± 1.50b	28.27 ± 0.87b	0.17 ± 0.01a	100.46 ± 6.73c	44.73 ± 2.29c	7.33 ± 0.24d	0.53 ± 0.30a
Means		375.92	96.43	44.12	0.22	213.25	67.88	12.53	0.43
LSD _0.05_		115.36	24.19	10.89	0.17	77.69	8.30	1.68	0.54
Xiangyaxianzhan	0	202.74 ± 29.77b	121.53 ± 0.33ab	27.40 ± 1.91a	0.50 ± 0.03a	129.22 ± 3.57a	55.47 ± 2.63ab	6.40 ± 0.50b	0.70 ± 0.49a
	400	268.95 ± 18.82a	129.54 ± 1.21ab	28.27 ± 0.71a	0.72 ± 0.13a	136.07 ± 8.89a	56.60 ± 1.75a	8.67 ± 0.13a	1.15 ± 0.55a
	800	240.64 ± 12.66ab	135.80 ± 13.36a	28.68 ± 0.52a	0.97 ± 0.32a	143.84 ± 8.81a	50.40 ± 1.29bc	10.07 ± 0.58a	1.41 ± 0.82a
	1,200	229.68 ± 11.74ab	108.00 ± 0.42b	23.07 ± 0.24b	1.17 ± 0.27a	124.20 ± 6.34a	46.33 ± 0.44c	6.93 ± 0.59b	0.42 ± 0.12a
Means		235.50	123.72	26.86	0.84	133.33	52.20	8.02	0.92
LSD _0.05_		63.96	21.88	3.45	0.72	23.60	5.61	1.60	1.81
Basmati-385	0	115.53 ± 5.94a	65.53 ± 0.24b	24.80 ± 0.50a	0.17 ± 0.07a	109.59 ± 3.95b	55.40 ± 1.29b	13.77 ± 0.83b	0.34 ± 0.12a
	400	157.08 ± 33.22a	71.11 ± 0.67b	25.87 ± 1.35a	0.20 ± 0.02a	163.93 ± 7.31a	62.93 ± 2.01a	18.60 ± 1.93a	0.80 ± 0.25a
	800	172.60 ± 10.46a	72.13 ± 5.62b	26.13 ± 2.10a	0.26 ± 0.04a	189.95 ± 0.46a	61.13 ± 1.45a	19.60 ± 0.42a	0.72 ± 0.08a
	1,200	158.45 ± 13.57a	104.67 ± 0.79a	18.33 ± 0.41b	0.28 ± 0.09a	168.95 ± 14.27a	65.67 ± 1.29a	19.93 ± 1.16a	0.85 ± 0.21a
Means		150.92	78.36	23.78	0.23	158.11	61.28	17.98	0.68
LSD _0.05_		61.71	9.33	4.21	0.20	26.93	5.01	3.97	0.58

*Values are the means of three independent determinations ± SE. Values with different lower case letters differ significantly at P < 0.05 according to least significant difference test. HS, Heading stage; MS, Maturity stage; SOD, Superoxide dismutase, POD, Peroxidases, CAT, Catalases; APX, Ascorbate peroxidases*.

### GSH contents and reduced to oxidized GSH (GSSG), total glutathione (GSH+GSSG) and GSH/GSH ratio

In response to Pb, all rice cultivars showed substantial changes in GSH, GSSG, and the total glutathione (GSH+GSSG) at both HS and MS at different Pb-treatments. For instance, at HS, an increase of 67.47, 45.18, and 86.78% in GSH, 88.34, 57.28, and 117.54% in GSSG and 69.16, 39.74, and 88.59% in total glutathione (GSH+GSSG) levels were recorded in Meixiangzhan-2, Xiangyaxianzhan, and Basmati-385, respectively as compared with control. Moreover, at MS, the levels of GSH and GSSG and total GSH+GSSG were remained statistically similar (*P* > 0.05) for Meixiangzhan-2, and only GSSG for Xiangyaxianzhan for all the treatments including control while GSH and total GSH+GSSG decreased with higher levels of Pb toxicity. The GSH, total GSH+GSSG were found highest at 1,200 ppm and GSSG at 800 ppm with a percentage increase of 26.70, 21.49, and 91.37%, respectively. In addition, the ratio of GSH/GSSG was found statistically similar (*P* > 0.05) for Meixiangzhan-2 and Basmati-385, and not for Xiangyaxianzhan (highest at 1,200 ppm) at HS. At MS, no significant changes were observed for GSH/GSSG in Meixiangzhan-2 and Xiangyaxianzhan while varied significantly (*P* < 0.05) in Basmati-385 with an increase from 0 to 800 ppm and then decreased with further increase in Pb-toxicity. Overall, the GSH, GSSG and total GSH+GSSG were higher at MS than HS while the ratio of GSH/GSSG was found higher at HS than MS in all rice cultivars (Table 4).

**Table 4:** **Effect of different Pb concentrations on non-enzymatic anti-oxidants of three scented rice cultivars at heading and maturity stages**.

**Rice cultivars**	**Pb (ppm)**	**HS**				**MS**			
		**GSH**	**GSSG**	**GSH+GSSG**	**GSH/GSSG**	**GSH**	**GSSG**	**GSH+GSSG**	**GSH/GSSG**
		**μmol g**^**−1**^ **FW**				**μmol g**^**−1**^ **FW**			
Meixiangzhan-2	0	241.00 ± 18.70c	21.24 ± 2.49b	262.24 ± 19.29c	11.66 ± 1.53a	429.65 ± 62.94a	61.43 ± 10.01a	491.08 ± 55.90a	7.75 ± 2.52a
	400	232.73 ± 1.79c	21.86 ± 2.28b	254.59 ± 3.74c	10.89 ± 1.19a	369.72 ± 17.25a	56.43 ± 15.52a	426.15 ± 28.11a	7.38 ± 1.50a
	800	324.03 ± 4.74b	36.19 ± 2.12a	360.22 ± 6.13b	9.01 ± 0.48a	318.66 ± 54.62a	82.14 ± 5.36a	400.80 ± 59.13a	3.84 ± 0.47a
	1,200	403.61 ± 12.70a	40.00 ± 3.38a	443.61 ± 13.16a	10.23 ± 0.93a	379.53 ± 31.06a	59.29 ± 6.94a	438.81 ± 26.01a	6.72 ± 1.40a
Means		300.34	29.82	330.17	10.45	374.39	64.82	439.21	6.42
LSD _0.05_		37.78	8.51	39.84	3.59	147.72	33.33	146.64	5.35
Xiangyaxianzhan	0	227.36 ± 15.91c	24.52 ± 1.86b	251.88 ± 14.80b	9.45 ± 1.30bs	424.28 ± 38.73a	55.48 ± 3.90a	479.76 ± 35.27a	7.83 ± 1.31a
	400	281.07 ± 15.91b	27.14 ± 1.80b	308.21 ± 17.57a	10.37 ± 0.29b	368.79 ± 23.68*a*b	51.19 ± 1.04a	419.98 ± 22.65*a*b	7.23 ± 0.61a
	800	295.39 ± 9.30*a*b	38.57 ± 1.65a	333.96 ± 7.65a	7.71 ± 0.57b	374.16 ± 18.31*a*b	41.19 ± 7.38a	415.35 ± 15.22*a*b	9.90 ± 2.27a
	1,200	330.08 ± 14.23a	21.91 ± 3.43b	351.99 ± 16.49a	15.64 ± 1.85a	327.61 ± 11.18b	53.10 ± 2.93a	380.71 ± 13.64b	6.19 ± 0.22a
Means		283.48	28.04	311.51	11.24	373.71	50.24	423.95	7.79
LSD _0.05_		45.98	7.51	47.78	3.84	81.87	14.52	76.04	4.40
Basmati-385	0	216.62 ± 8.95c	13.57 ± 1.89b	230.19 ± 8.28c	16.26 ± 1.09a	341.93 ± 20.65b	27.62 ± 1.04b	392.65 ± 21.04*a*b	6.92 ± 0.96b
	400	288.23 ± 39.26*b*c	14.05 ± 6.32b	302.27 ± 37.26bc	22.67 ± 2.65a	368.18 ± 21.69*a*b	50.71 ± 5.41a	421.04 ± 16.79*a*b	7.31 ± 1.24b
	800	311.50 ± 16.32b	21.67 ± 5.12*a*b	333.17 ± 11.41b	16.66 ± 4.95a	331.19 ± 18.95b	52.86 ± 7.30a	358.81 ± 17.96b	12.08 ± 1.13a
	1,200	404.59 ± 22.97a	29.52 ± 0.26a	434.12 ± 24.18a	13.69 ± 1.26a	433.24 ± 35.67a	43.81 ± 7.08*a*b	477.04 ± 40.51a	10.26 ± 1.23ab
Means		305.24	19.70	324.94	17.32	368.64	43.75	412.39	9.14
LSD _0.05_		80.14	9.56	75.99	13.63	81.99	18.85	84.55	3.74

*Values are the means of three independent determinations ± SE. Values with different lower case letters differ significantly at P < 0.05 according to least significant difference test. HS, Heading stage; MS, Maturity stage; GSH, Reduced glutathione; GSSG, oxidized glutathione*.

### Yield and grain quality related attributes

All Pb-treatments caused considerable reduction in yield and related attributes and resulted in deterioration of grain quality in all rice cultivars (Tables 5, 6), in dose dependent manner. Pb-stress reduced 15.70, 27.03, and 15.63% total tillers/pot, 24.10, 33.33, and 20.27% productive tillers/pot, 19.66, 21.02, and 12.03% grains/ panicle, 9.48, 15.36, and 11.03% 1,000-grain weight, 58.05, 69.12, and 46.27% grain yield pot^−1^, and 44.24, 61.11, and 39.30% harvest index while 111.95, 230.01, and 55.31% increase in sterility percentage were recorded in Meixiangzhan-2, Xiangyaxianzhan and Basmati-385, respectively. In addition, tillers/pot and 1,000-grain weight for Meixiangzhan-2 while productive tillers/pot, grains/panicle and sterility percentage for Basmati-385 were remained statistically similar (*P* > 0.05) (Table 5).

**Table 5:** **Effect of different Pb concentrations yield and yield related components of three scented rice cultivars**.

**Rice cultivars**	**Pb (ppm)**	**Tillers/pot**	**Productive Tillers/pot**	**Grains/panicle**	**Sterility percentage (%)**	**1,000-grain weight (g)**	**Grain yield/pot (g)**	**Harvest Index**
Meixiangzhan-2	0	40.41 ± 2.19a	27.72 ± 1.46a	162.44 ± 6.72a	17.65 ± 0.64c	19.70 ± 0.67a	72.93 ± 4.65a	61.94 ± 8.24a
	400	38.08 ± 3.22a	26.72 ± 0.33a	152.11 ± 8.99ab	21.09 ± 2.78bc	18.20 ± 1.37a	58.91 ± 7.88a	50.79 ± 9.81ab
	800	37.07 ± 3.06a	22.04 ± 0.58b	138.55 ± 3.43bc	24.88 ± 0.25b	18.09 ± 0.79a	41.37 ± 0.45b	40.11 ± 3.53ab
	1,200	34.07 ± 1.00a	21.04 ± 0.58b	130.50 ± 1.72c	37.41 ± 2.32a	17.84 ± 0.45a	30.82 ± 2.72b	34.54 ± 6.49b
Means		37.41	24.38	145.90	25.26	18.46	51.01	46.85
LSD _0.05_		8.24	2.78	19.35	6.02	2.90	15.58	24.11
Xiangyaxianzhan	0	37.07 ± 3.06a	23.05 ± 2.09a	174.46 ± 4.40a	11.78 ± 1.22c	21.57 ± 0.69a	76.97 ± 9.43a	68.40 ± 11.48a
	400	33.40 ± 0.33ab	19.37 ± 0.88ab	164.07 ± 11.41a	23.18 ± 2.15b	19.96 ± 0.44ab	48.83 ± 4.75b	45.20 ± 6.39ab
	800	29.06 ± 1.00bc	17.03 ± 0.58b	154.37 ± 5.14ab	33.74 ± 3.59a	18.45 ± 0.25bc	32.09 ± 1.88bc	31.54 ± 2.72b
	1,200	27.05 ± 1.74c	15.36 ± 2.34b	137.78 ± 3.53b	38.87 ± 2.15a	18.26 ± 0.52c	23.46 ± 3.36c	26.60 ± 7.15*a*b
Means		31.65	18.70	157.67	26.89	19.56	45.34	42.94
LSD _0.05_		5.99	5.39	22.39	7.93	1.63	18.33	24.79
Basmati-385	0	32.06 ± 1.53a	24.72 ± 2.61a	157.80 ± 26.03a	20.09 ± 4.29a	19.88 ± 0.47a	59.95 ± 5.38a	58.19 ± 6.31a
	400	30.73 ± 1.86ab	24.05 ± 2.52a	151.49 ± 23.94a	21.57 ± 5.03a	19.52 ± 0.75ab	53.78 ± 3.53ab	55.85 ± 3.20a
	800	30.06 ± 1.53ab	21.38 ± 2.03a	140.70 ± 8.10a	25.51 ± 1.50a	19.27 ± 0.27ab	42.65 ± 0.96bc	44.24 ± 4.98ab
	1,200	27.05 ± 0.00b	19.71 ± 1.20a	138.82 ± 5.49a	31.19 ± 3.61a	17.69 ± 0.94b	33.12 ± 2.47c	35.33 ± 1.58b
Means		29.98	22.47	147.20	24.59	19.09	47.38	48.40
LSD _0.05_		4.65	7.06	59.82	12.52	2.14	11.38	14.34

*Values are the means of three independent determinations ± SE. Values with different lower case letters differ significantly at P < 0.05 according to least significant difference test*.

**Table 6:** **Effect of different Pb concentrations on grain quality attributes of three scented rice cultivars**.

**Rice cultivars**	**Pb (ppm)**	**Brown rice rate (%)**	**Milling recovery (%)**	**Milling degree (%)**	**Head rice rate (%)**	**Chalkiness rate (%)**	**Chalkiness degree (%)**	**Moisture contents (%)**	**Amylose contents (%)**	**Protein contents (%)**	**Grain alkali**
Meixiangzhan-2	0	76.41 ± 3.14a	62.73 ± 1.28a	81.91 ± 0.56a	75.93 ± 2.13a	30.37 ± 1.32d	10.44 ± 0.34c	12.57 ± 0.03a	18.70 ± 0.42b	7.57 ± 0.03a	6.33 ± 0.09a
	400	75.84 ± 1.70a	61.85 ± 0.52ab	81.23 ± 1.10a	74.07 ± 4.21a	35.40 ± 2.30c	12.29 ± 0.64b	13.60 ± 0.95a	20.73 ± 1.04a	6.67 ± 0.03c	6.23 ± 0.07a
	800	75.40 ± 2.16a	61.45 ± 0.63ab	83.03 ± 0.11a	59.70 ± 3.46a	40.43 ± 0.57b	12.55 ± 0.33ab	12.80 ± 0.06a	19.10 ± 0.46ab	6.50 ± 0.06d	6.20 ± 0.06a
	1,200	71.25 ± 1.10a	58.35 ± 1.54b	76.38 ± 0.92b	54.13 ± 3.44a	47.50 ± 1.32a	13.90 ± 0.47a	12.50 ± 0.06a	20.90 ± 0.25a	7.20 ± 0.06b	6.20 ± 0.06a
Means		74.73	61.10	80.64	65.96	38.43	12.30	12.87	19.86	6.99	6.24
LSD _0.05_		7.04	3.53	2.52	23.92	4.92	1.51	1.55	2.01	0.15	0.22
Xiangyaxianzhan	0	77.87 ± 1.90a	65.45 ± 1.59a	86.33 ± 0.70a	67.10 ± 3.94a	25.74 ± 1.96b	11.54 ± 0.71b	12.80 ± 0.06a	17.57 ± 0.27b	6.33 ± 0.03c	6.20 ± 0.10a
	400	75.78 ± 2.60ab	64.23 ± 0.46a	82.86 ± 0.86b	63.87 ± 3.91a	30.68 ± 0.66b	12.95 ± 0.58b	12.80 ± 0.05a	18.30 ± 0.31b	6.43 ± 0.03c	6.20 ± 0.06a
	800	75.50 ± 1.74ab	62.35 ± 1.00a	80.23 ± 0.70b	58.80 ± 6.31ab	31.67 ± 2.40b	13.73 ± 0.91b	12.70 ± 0.06ab	17.70 ± 0.40b	7.50 ± 0.06a	6.00 ± 0.06a
	1,200	70.04 ± 0.98b	58.17 ± 0.97b	80.70 ± 0.98b	47.05 ± 4.00b	41.00 ± 2.31a	18.54 ± 0.59a	12.60 ± 0.04b	22.73 ± 1.00a	7.10 ± 0.06b	5.97 ± 0.28a
Means		74.80	62.55	82.53	59.21	32.27	14.19	12.73	19.08	6.84	6.09
LSD _0.05_		6.17	3.53	2.67	15.17	6.40	2.32	0.19	1.88	0.15	0.51
Basmati-385	0	78.18 ± 1.40a	64.45 ± 0.63a	83.85 ± 0.97a	69.52 ± 2.31a	27.71 ± 0.89b	7.14 ± 0.49c	12.73 ± 0.07a	17.97 ± 0.35c	6.43 ± 0.03a	6.07 ± 0.07a
	400	76.77 ± 0.40ab	62.06 ± 0.43b	79.84 ± 1.47b	67.03 ± 2.09ab	29.93 ± 0.58b	9.19 ± 0.40b	12.67 ± 0.06ab	18.33 ± 0.27*b*c	5.80 ± 0.06c	5.87 ± 0.03b
	800	74.24 ± 0.49b	61.38 ± 0.66*b*c	83.10 ± 1.51ab	60.00 ± 4.16b	31.78 ± 0.78ab	9.96 ± 0.10ab	12.50 ± 0.09b	19.27 ± 0.19ab	6.00 ± 0.06*b*c	5.70 ± 0.06bc
	1,200	74.38 ± 0.30b	60.24 ± 0.46c	84.49 ± 0.82a	59.19 ± 2.63b	35.67 ± 3.18a	10.53 ± 0.33a	12.50 ± 0.06b	20.27 ± 0.56a	6.07 ± 0.09b	5.60 ± 0.06c
Means		75.89	62.03	82.82	63.94	31.27	9.21	12.60	18.96	6.08	5.81
LSD _0.05_		2.55	1.81	4.01	9.50	5.61	1.17	0.22	1.21	0.20	0.18

*Values are the means of three independent determinations ± SE. Values with different lower case letters differ significantly at P < 0.05 according to least significant difference test*.

Pb toxicity reduced brown rice rate (6.76, 10.06, and 4.87%), milling recovery (6.99, 11.12, and 6.53%), milling degree (6.76, 4.18, and 4.78%), head rice rate (28.71, 29.88, and 14.87%), while increased chalkiness rate (56.42, 59.30, and 28.72%) and rice chalkiness degree (33.17, 60.64, and 47.48%) in Meixiangzhan-2, Xiangyaxianzhan and Basmati-385, respectively. Moreover, moisture contents for Meixiangzhan-2 and grain alkali for Meixiangzhan-2 and Xiangyaxianzhan were found statistically similar (*P* > 0.05) while reduced significantly (*P* < 0.05) in Basmati-385. Further, reduced grain moisture contents i.e., 1.56 and 1.83% for Xiangyaxianzhan and Basmati-385, and 14.10, 18.42, and 9.84% in grain protein for Meixiangzhan-2, Xiangyaxianzhan and Basmati-385, respectively were recorded. Additionally, grain amylose contents increased under Pb stress conditions, and the values of increase percentage were reached at 11.76, 29.41, and 12.80% for Meixiangzhan-2, Xiangyaxianzhan and Basmati-385, respectively (Table 6).

### Correlation analyses among yield and yield contributing factors under Pb toxicity

**Figure 1:**
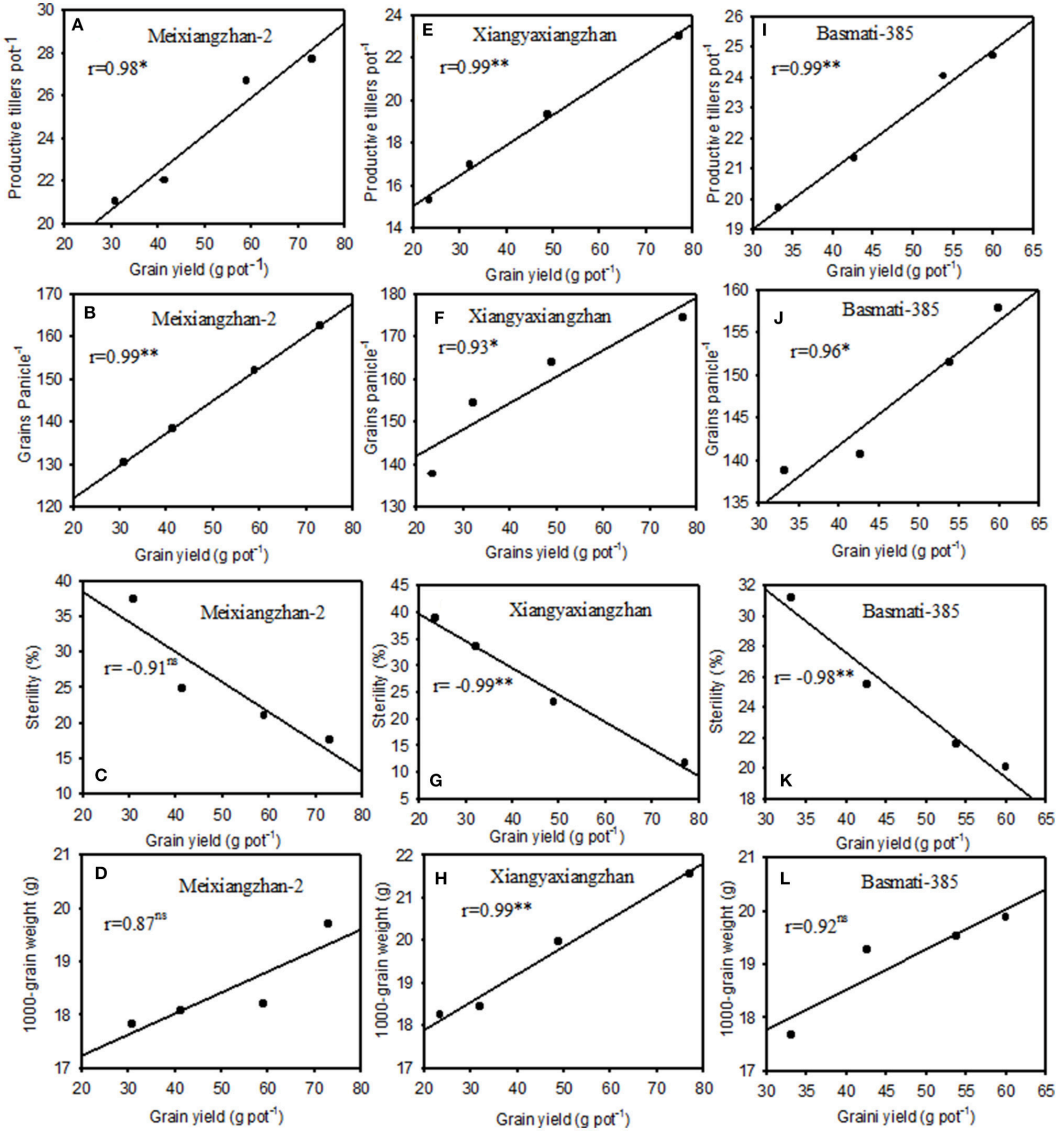
**Relationships between grain yield per pot with productive tiller pot^**−1**^, grains panicle^**−1**^, sterility (%), and 1,000-grain weight for Meixiangzha-2 (A–D)**, for Xiangyaxiangzhan **(E–H)**, and for Basmati-385 **(I–L)** under different Pb concentrations using polynomial linear regression analyses. ^*^Significant at *P* < 0.05, ^**^Significant at *P* < 0.05, ns, non-significant.

Significant correlations (*P* < 0.05) were recorded among grain yield with yield related attributes in all rice cultivars under Pb-treatments. Significant and positive correlations between rice yield with productive tillers/pot (*r* = 0.98, 0.99, and 0.99) and grains per panicle (0.99, 0.93, and 0.96) were observed in Meixiangzhan-2, Xiangyaxianzhan and Basmati-385, respectively while significant but negative with sterility percentage (*r* = −0.99 and −0.98) for Xiangyaxianzhan and Basmati-385, respectively; however relationships of grain yield with sterility percentage were remained non-significant (*P* > 0.05) for Meixiangzhan-2. Furthermore, the relationships of rice grain yield with 1,000-grain weight were only significant for Xiangyaxianzhan (*r* = 0.99), while remained non-significant (*P* > 0.05) for Meixiangzhan-2 and Basmati-385 under Pb-toxicity (Figure 1).

### Pb uptake and percentage accumulation in different plant parts

**Figure 2:**
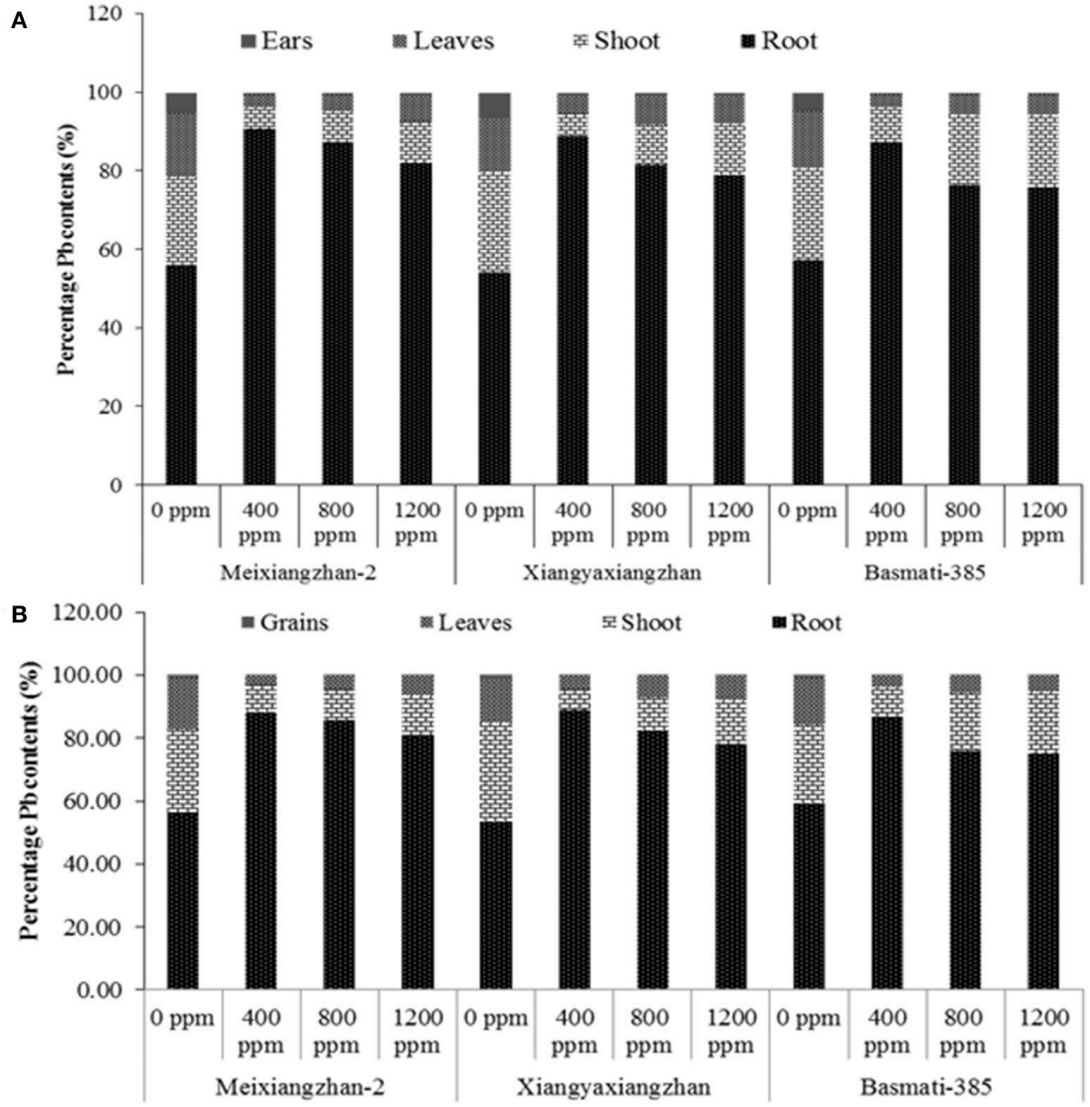
**Percentage distribution of Pb contents in (A)** root, stems, leaves and ears at heading stage (HS) and **(B)** root, stems, leaves and grains at maturity stage (MS) in Meixiangzhan-2, Xiangyaxiangzhan and Basmati-385. Each vertical column at each level of Pb represents the respective proportions of Pb contents in all parts under the same Pb level for a given rice cultivar.

The percentage Pb contents within plants parts are increased with an increase in the concentration of Pb applied. The roots stored the maximum proportions of Pb contents in all rice cultivars at all Pb-treatments. At HS, the root Pb proportions were remained up to 9.98, 6.82, and 4.65-fold higher for Meixiangzhan-2, 8.08, 4.44, and 3.76-fold higher for Xiangyaxianzhan and 6.78, 3.22, 3.13-fold higher for Basmati-385 than the combined/total Pb proportions (%) in stems, leaves and Meixiangzhan-2, Xiangyaxianzhan and Basmati-385, respectively at 400, 800, and 1,200 ppm soil Pb levels, respectively; however, the values were decreased as soil Pb level increased. Further stems accumulated 1.95, 1.87, and 3.76-fold higher Pb contents (%) than leaves and 13.60, 15.83, and 35.68-fold higher than ear Pb contents while the leaves Pb contents were remained 8.55, 8.45, and 9.49-fold higher than ears of Meixiangzhan-2, Xiangyaxianzhan and Basmati-385, respectively. At MS, root stored 7.53, 7.86, and 6.39-fold of Pb contents than all other plant parts whereas the stems Pb contents were 2.69, 1.95, and 3.78 times higher Pb stored than leaves of Meixiangzhan-2, Xiangyaxianzhan and Basmati-385, respectively. Moreover, grain Pb contents were 29.88, 31.99, and 32.64-fold lower than leaves Pb contents in Meixiangzhan-2, Xiangyaxianzhan and Basmati-385, respectively (Figure 2). Means across all three cultivars showed that the pattern of Pb accumulation in different plant parts as follows (mean values, mg kg^−1^): root (1,892, 2,916, and 4,606) > stem (140, 420, and 817) > leaves (76, 191, and 362) > ears (16, 26, and 41) at HS whist root (2,163, 3,125, and 4,763) > stem (470, 488, and 957) > leaves (216, 215, and 389) > grains (4.04, 8.44, and 12.39) at 400, 800, and 1,200 ppm Pb concentrations, respectively at MS. Hence, the Pb contents were decreased along the plant from root to ears/grains in all rice cultivars.

## Discussion

Present study revealed the toxic effects of Pb on photosynthetic pigments, plant metabolism, yield and quality and its bio-accumulation in three different scented rice cultivars. Pb-toxicity severely affected photosynthetic pigments in Meixiangzhan-2, Xiangyaxianzhan and Basmati-385 and the effects were become more intense with an increase in soil Pb-concentration at both HS and MS. (Table 1). Previous studies demonstrated the leaf chlorosis and/or leaf discoloration and chlorophyll destruction are the consequences of Pb-toxicity (Ernst et al., 2000; Srivastava et al., 2014), as depicted from our studies. Under Pb stress, the central Mg^2+^ is replaced by Pb^2+^, thus impaired chlorophyll molecules, caused chlorophyll ultrastructural changes and inhibit chlorophyll biosynthesis (Kupper et al., 1996). Further, Anjum et al. (2016c) also observed significant reduction in Chl a, Chl b, and total Chl a+b in maize facing individual and combined metal toxicity (Al and Cr). Increased chlorophyllase activities under metal toxicity often resulted destruction of chlorophyll production and carotenoids and/or may undergo to photo-oxidation (Luna et al., 1994; Gill et al., 2015).

Pb toxicity led to destructive changes at both HS and MS in scented rice in terms of H_2_O_2_ production, lipid peroxidation (MDA production) and membrane damage (electrolyte leakage) and the rate of destruction was highest in Xiangyaxianzhan followed by Meixiangzhan-2 and Basmati-385 (Table 2). Exposure of Pb stress in rice might resulted in over-production of reactive oxygen species (ROS) which oxidize essential cellular structures and cell membranes. MDA production due to peroxidation of polyunsaturated fatty acids is a key indicator of oxidative stress, while leaf leachates provide an estimated degree of membrane damage. Production of H_2_O_2_ up to a certain level can play a signaling role to activate the stress-tolerant related genes under abiotic stresses; however, its high rates of production and excess accumulation would lead to severe consequences in plants (Prasad et al., 1994). Pb-induced higher rates of H_2_O_2_ production were also reported by Chen et al. (2007) in 18-d old rice seedlings. Excessive rate of degradation in Xiangyaxianzhan and Meixiangzhan-2 would probably due to high-rate activities of ROS at higher Pb-levels which resulted in loss in membrane integrity and cellular damage than Basmati-385, and showed a Pb-sensitive behavior. Moreover, less ROS-induced injury in Basmati-385 than other two rice cultivars possibly due to reduced rate of production or its resilient abilities to scavenge ROS. Enhanced oxidative damage in terms of ROS production in Pb-sensitive rice seedlings were also described by Verma and Dubey (2003). Similarly, higher rates of oxidative damage and lipid peroxidation were also found in Ni-sensitive rice seedlings “Pant-12” under elevated Ni-levels (Maheshwari and Dubey, 2009).

Osmo-regulation through accumulation of various osmolytes is an important plant defensive response against multiple abiotic stresses (Farooq et al., 2009; Pourrut et al., 2011; Fahad et al., 2016b). In this study, Pb toxicity changed the production/accumulation of protein, proline and soluble sugars; however the rate of production was varied among all three rice cultivars and Pb-toxicity levels. Among three rice cultivars, Xiangyaxianzhan was found least effective regarding osmo-regulation under Pb-toxicity (Table 2). Biosynthesis and relative accumulation of these osmolytes are well-recognized due to a wide range of their functions, i.e., protection and stabilization of cellular membranes, protection of several enzymes, act as osmoticum for turgor maintenance, and scavenging roles against ROS (e.g., proline) in plants under heavy-metal stress (Rodriguez et al., 2012; Ali et al., 2014; Ashraf et al., 2015). Our results further revealed that the production and accumulation of these osmolytes may be reduced with higher rates of Pb-toxicity (Table 1). These dynamics in osmolyte accumulation possibly depends on comparative production and detoxification of ROS within plant cells/tissues, which can be explained that at higher Pb-levels, the production rates and activities of ROS might be too high to be quenched or the rate of osmolyte are not increased with respective to the ROS generated. Dynamic accumulation and roles of these osmolytes under Pb stress were previously reported by Chatterjee et al. (2004) and Piotrowska et al. (2009) who concluded that these osmolytes and protein pools may decrease at high Pb-levels, which may lead to severe oxidative stress. Furthermore, utilization of proteins in Pb detoxification and Pb-induced reduction in free amino acids may disturb N-metabolism in plants (Chatterjee et al., 2004; Gupta et al., 2009). However, increased proline contents which may play a significant role in Pb tolerance under Pb toxicity were recorded in *Cassia angustifolia* Vahl (Qureshi et al., 2007). Hence, any decrease in the concentrations of osmolytes would affect the Pb-tolerance mechanism in rice.

We have observed significant variations among defense system in terms of SOD, POD, CAT, and APX activities as enzymatic and accumulation of GSH, GSSG and total GSH+GSSG as non-enzymatic anti-oxidants in Meixiangzhan-2, Xiangyaxianzhan and Basmati-385 at both sampling stages; however, the activities of enzymatic and non-enzymatic anti-oxidants were Pb-concentration dependent. Moreover, at initial levels of Pb toxicity, the enzymatic anti-oxidants activities increased, but then decreased abruptly with further increase in Pb toxicity; however, the values of percentage decrease were recorded as Xiangyaxianzhan > Meixiangzhan-2 > Basmati-385 (Tables 3, 4). Upon lead exposure, plants activate their anti-oxidative defense mechanism to quench Pb-generated ROS to minimize oxidative stress; however, the activities of these enzymes can be reduced with metal intensification. Normally, increase or decrease in enzymatic activities depends on plant genotype, extent of stress to which plants are exposed, metal concentration, plant age, form of metal in rhizosphere, and duration of metal exposure (Islam et al., 2008; Singh et al., 2010). A specific Pb level might be inhibitory for one enzyme but promotive for the others; however these dynamics in their activities vary plant to plant. Some researchers declared Pb-induced increase in anti-oxidative defense system (Choudhury and Panda, 2004; Reddy et al., 2005) while other found significant reductions in their activities at higher Pb levels (Mishra et al., 2006; Chen et al., 2007). SOD, a metallo-enzyme, detoxifies ${\text{O}}_{2}^{-}$ to H_2_O_2_ and O_2_, hence play a crucial role to cleanse the ROS while CAT and APX are also key enzyme involved in the catabolism of H_2_O_2_ to H_2_O and O_2_ (Garg and Manchanda, 2009). Activities of POD provide protection to cell membranes through detoxification of free radicals that normally cause lipid peroxidation (Radotic et al., 2000). In our study, overall activities of these anti-oxidants were remain higher from low to medium Pb levels (400–800 ppm), and then reduced with further increase in Pb concentration; however, such a decrease was low in Basmati-385, which shows its ability to maintain anti-oxidative defense system more efficiently than other two rice cultivars. The possible reason of enhanced activities at lower Pb concentrations could be due to high detoxification rates of ROS produced in the plant tissues, however, at higher rate (1,200 ppm as in our case), the production and activity rate of ROS may be too high that the ROS scavenging activities of anti-oxidants and/or the production rate of these enzymes do not remain high enough to quench ROS at high-Pb concentrations. Both increased and decreased anti-oxidant activities under different heavy metals were previously reported (Guo et al., 2007; Anjum et al., 2016c).

Both GSH and GSSG are the most abundant non-enzymatic anti-oxidants in plants and also provide protection in plant cells from ROS-generated free radicals and serve as redox buffer (Sharma and Dietz, 2009). Our results showed a significant increase in both GSH and GSSG as well as total GSH+GSSG contents in all rice cultivars under Pb-stress, but such an increment (on percentage basis) were higher in Basmati-385 followed by Meixiangzhan-2 and Xiangyaxianzhan (Table 4). GSH has a direct role in Pb-detoxification and an essential component of Halliwell-Asada pathway where inter-converted into GSSG and GSH by two enzymes i.e., glutathione reductase (GR) and dehydroascorbate reductase (DHAR). Increased rates of GSH under Pb stress showed their ability to detoxify ROS by involving in H_2_O_2_-scavenging mechanism (Piechalak et al., 2002). Further, higher GSH/GSSG ratio in Basmati-385 was attributed to higher anti-oxidative capacity of this cultivar under Pb stress; while decline in GSH/GSSG ratio at MS than HS representing reduced anti-oxidant activities at MS. Contrary to our results, Srivastava et al. (2014) found decline in GSH contents in rice seedlings under interactive Pb and Cd stress, while both GSH and GSSG contents were increased in *Brassica napus* under Pb stress (Ali et al., 2014). Recent results of Mostofa et al. (2015) are in accordance with our studies who found a considerable increase in glutathione contents in rice seedlings under Cu stress.

Yield and quality components of all rice cultivars differentially affected by Pb-toxicity; however the reductions were more apparent in Xiangyaxiangzhan followed by Meixiangzhan-2 whilst Basmati-385 was least affected by Pb-stress (Tables 5, 6). External environment and genotypes interactions as well as crop management are the factors that are directly related to the yield formation and quality attributes of aromatic rice. Reductions in yield components under Pb toxicity would lead to reduced grain yield and harvest indices of all rice cultivars with maximum yield loss in Xiangyaxiangzhan. Previously, Pb concentration-related changes in rice yields were also observed, for example, soil Pb concentration at 1,000 mg kg^−1^ resulted in about 12% rice yield reduction (Gu et al., 1989), improved up to 2.54% (at 100 mg kg^−1^) and then decreased to 4.61% as soil Pb concentrations increased to 300 mg kg^−1^ of soil (Wang and Wu, 1997). Furthermore, Xie and Huang (1994) argued that low Pb levels could enhance rice tillering ability while would lead to significant reductions as soil Pb concentrations increased. Plant external factors led to significant changes in rice quality attributes especially in aromatic rice cultivars. Significant reductions in rice quality attributes i.e., brown and milled rice rates as well as head milled rice rate were found in two rice cultivars, “Yuxiangyouzhan” and “Tianyou998” exposed to individual (Pb and Cd) and combined metal (Pb+Cd) toxicity (Wang et al., 2010). Influences of external factors on rice quality attributes including rice chalkiness, amylose and grain protein contents grain amylose were also previously reported (Li et al., 2016; Mo et al., 2016). In addition, we have observed significant relationships of rice grain yield with most of the yield related attributes in all rice cultivars (Figure 1). Our results also corroborates with the findings of Liu et al. (2003) who concluded that rice yield components could have significant relationship with paddy yield under Pb toxic conditions; and any changes in yield components would have an ultimate effect of grain yield of rice.

In our study, all three aromatic rice cultivars accumulated different proportions of Pb contents in different plant parts; nonetheless the trend for Pb distribution proportion (from minimum to maximum) for all rice cultivars were recorded as: grain (at MS) < ear (at PH) < leaves < stems < root. Plant Pb contents were also affected by soil Pb-concentration (Figure 2). Higher Pb contents in above ground plant parts impart rice morphology and physiology, and would thus result in yield penalty. More transportation of Pb to grain may lead to quality deterioration and would raise grain quality issues. Among three rice cultivars, highest losses in Xiangyaxiangzhan may be related to higher Pb contents accumulated in above ground plant parts than Meixiangzhan-2 and Basmati-385. Plant characteristics and presence of Pb form in the soil solution would normally determine the fate of Pb in soil-plant-Pb interaction (Stoltz and Greger, 2002). Soil Pb concentration may possibly increase its accumulation in edible plant parts, regardless its mobilization rate from soil to plant (Li et al., 2007). Our results of increased soil Pb concentrations related increase in grain Pb contents are in accordance with Feng et al. (2011). On the other hand, Liu et al. (2015) reported that soil Pb contents and its respective accumulation in the grains has no one-to-one correlations and is not the only determinant for grain Pb accumulation in rice which suggests not only the soil Pb contents but also various other factors may also involve in accumulation and transportation of Pb in the plants. Hence, Pb transformation, transportation and accumulation mechanisms in soil-plant system are quite complex and would largely depends on plant characters, soil physicochemical properties and biological activities within the soil (Zhao et al., 2010). However, Pb uptake and its internal distribution with different plant parts would determine sensitivity and tolerance index in rice (Rout et al., 2001).

## Conclusions

This study concluded that Pb toxicity may have severe consequences on rice yield and quality characters by changing its internal physio-biochemical mechanisms and inhibiting biosynthesis of photosynthetic pigments in scented rice. Among studied rice cultivars Basmati-358, Meixiangzhan-2 and Xiangyaxiangzhan proved tolerant, medium, sensitive to lead (Pb) with respect to percentage yield reduction and Pb accumulation behavior (higher in edible plant parts than other two rice cultivars). Moreover, higher Pb concentrations in the soil led to increased Pb proportions of Pb contents in grains of all rice cultivars; but the accumulation pattern from minimum to maximum was observed as: grain (at MS) < ear (at HS) < leaves < stems < root. In our case, although all Pb levels damaged the aromatic rice cultivars while soil Pb level exceeding 800 ppm resulted in significant losses in all rice cultivars. Hence, soil Pb level beyond 800 ppm could be toxic for fragrant rice cultivars and could bring significant yield losses along with rice quality deterioration.

## Author contributions

UA, ZM, and XT designed the research. UA, AK, and QD performed the experiments and collected the data. UA, AK, and QD performed the traits investigated and laboratory analyses. UA analyzed the data and wrote the manuscript. SP, HT, and XT provided guidance during experimentation. All authors approved the final version of the manuscript.

### Conflict of interest statement

The authors declare that the research was conducted in the absence of any commercial or financial relationships that could be construed as a potential conflict of interest.

## References

[B1] Abrahams P. W. (2002). Soils: Their implications to human health. Sci. Total Environ. 291, 1–32. 10.1016/S0048-9697(01)01102-0 12150429

[B2] Aebi H. (1984). Catalase *in-vitro*. Meth. Enzymol. 105, 121–126. 10.1016/S0076-6879(84)05016-3 6727660

[B3] Ali B. Xu X. Gill R. A. Yang S. Ali S. Tahir M. (2014). Promotive role of 5-aminolevulinic acid on mineral nutrients and antioxidative defense system under lead toxicity in *Brassica napus*. Ind. Crop Prod. 52, 617–626. 10.1016/j.indcrop.2013.11.033

[B4] Anjum S. A. Ashraf U. Khan I. Tanveer M. Ali M. Hussain I. (2016b). Chromium and aluminum phytotoxicity in maize: morpho-physiological responses and metal uptake. Clean Soil Air Water 44, 1–10. 10.1002/clen.201500532

[B5] Anjum S. A. Ashraf U. Khan I. Tanveer M. Saleem M. F. Wang L. C. (2016a). Aluminum and chromium toxicity in maize: implications for agronomic attributes, net photosynthesis, physio-biochemical oscillations, and metal accumulation in different plant parts. Water Air Soil Pollut. 227, 326 10.1007/s11270-016-3013-x

[B6] Anjum S. A. Ashraf U. Saleem M. F. Wang L. (2016c). Chromium toxicity induced alterations in growth, photosynthesis, gas exchange attributes and yield formation in maize. Pak. J. Agri. Sci. 53, 751–757. 10.21162/PAKJAS/16.3824

[B7] Arnon D. T. (1949). Copper enzyme in isolated chloroplasts polyphenoloxidase in *Beta vulgaris*. Plant Physiol. 24, 1–15. 10.1104/pp.24.1.1 16654194 PMC437905

[B8] Ashraf U. Kanu A. S. Mo Z. W. Hussain S. Anjum S. A. Khan I.. (2015). Lead toxicity in rice; effects, mechanisms and mitigation strategies-a mini review. Environ. Sci. Pollut. Res. 22, 18318–18332. 10.1007/s11356-015-5463-x 26432270

[B9] Bates L. S. Waldren R. P. Teare I. D. (1973). Rapid determination of free proline for water-stress studies. Plant Soil 39, 205–207. 10.1007/BF00018060

[B10] Bradford M. N. (1976). A rapid and sensitive method for the quantitation of microgram quantities of protein utilizing the principle of protein-dye binding. Anal. Chem. 72, 248–254. 10.1016/0003-2697(76)90527-3 942051

[B11] Chatterjee C. Dube B. K. Sinha P. Srivastava P. (2004). Detrimental effects of lead phytotoxicity on growth, yield, and metabolism of rice. Commun. Soil Sci. Plant Anal. 35, 255–265. 10.1081/CSS-120027648

[B12] Chen J. Zhu C. Li L. Sun Z. Pan X. (2007). Effects of exogenous salicylic acid on growth and H_2_O_2_-metabolizing enzymes in rice seedlings under lead stress. J. Environ. Sci. 19, 44–49. 10.1016/S1001-0742(07)60007-2 17913152

[B13] Choudhury S. Panda S. (2004). Toxic effects, oxidative stress and ultrastructural changes in moss *Taxithelium nepalense* (Schwaegr.) Broth. under chromium and lead phytotoxicity. Water Air Soil Pollut. 167, 73–90. 10.1007/s11270-005-8682-9

[B14] Clemens S. (2006). Toxic metal accumulation, response to exposure and mechanism of tolerance in plants. Biochimie 88, 1707–1719. 10.1016/j.biochi.2006.07.003 16914250

[B15] Ernst W. H. O. Nielssen H. G. M. Ten Bookum W. M. (2000). Combination toxicology of metal-enriched soils: physiological responses of a Zn- and Cd-resistant ecotypes of *Silene vulgari*s on polymetallic soils. Environ. Exp. Bot. 43, 55–71. 10.1016/S0098-8472(99)00048-9

[B16] Fahad S. Hussain S. Saud S. Hassan S. Chauhan B. S. Khan F.. (2016a). Responses of rapid viscoanalyzer profile and other rice grain qualities to exogenously applied plant growth regulators under high day and high night temperatures. PLoS ONE 11:e0159590. 10.1371/journal.pone.0159590 27472200 PMC4966964

[B17] Fahad S. Hussain S. Saud S. Khan F. Hassan S. Nasim W. (2016b). Exogenously applied plant growth regulators affect heat-stressed rice pollens. J. Agron. Crop Sci. 202, 139–150. 10.1111/jac.12148

[B18] Farooq M. Wahid A. Kobayashi N. Fujita D. Basra S. M. A. (2009). Plant drought stress: effects, mechanisms and management. Agron. Sustain. Dev. 29, 185–212. 10.1051/agro:2008021

[B19] Feng J. Wang Y. Zha J. Zhu L. Bian X. Zhang W. (2011). Source attributions of heavy metals in rice plant along highway in Eastern China. J. Environ. Sci. 23, 1158–1164. 10.1016/S1001-0742(10)60529-3 22125909

[B20] Garg N. Manchanda G. (2009). ROS generation in plants: boon or bane? Plant Biosys. 143, 8–96. 10.1080/11263500802633626

[B21] Gill R. A. Zang L. Ali B. Farooq M. A. Cui P. Yang S.. (2015). Chromium-induced physio-chemical and ultrastructural changes in four cultivars of *Brassica napus* L. Chemosphere 120, 154–164. 10.1016/j.chemosphere.2014.06.029 25016339

[B22] Gu S. H. Zhu J. Z. Gu Z. L. (1989). Study on the critical lead content of red paddy soil. Agro. Environ. Prot. 8, 17–22.

[B23] Guo T. R. Zhang G. P. Zhang Y. H. (2007). Physiological changes in barley plants under combined toxicity of aluminum, copper and cadmium. Colloids Surf B. Biointerfaces 57, 182–188. 10.1016/j.colsurfb.2007.01.013 17344036

[B24] Gupta D. Nicoloso F. Schetinger M. Rossato L. Pereira L. Castro G.. (2009). Antioxidant defense mechanism in hydroponically grown Zea mays seedlings under moderate lead stress. J. Hazard. Mater. 172, 479–484. 10.1016/j.jhazmat.2009.06.141 19625122

[B25] Hodges D. M. DeLong J. M. Forney C. F. Prange R. K. (1999). Improving the thiobarbituric acid-reactive-substances assay for estimating lipid peroxidation in plant tissues containing anthocyanin and other interfering compounds. Planta 207, 604–611. 10.1007/s004250050524 28456836

[B26] Islam E. Liu D. Li T. Yang X. Jin X. Mahmood Q.. (2008). Effect of Pb toxicity on leaf growth, physiology and ultrastructure in the two ecotypes of *Elsholtzia argyi*. J. Hazard. Mater. 154, 914–926. 10.1016/j.jhazmat.2007.10.121 18162296

[B27] Kumar A. Prasad M. N. V. Sytar O. (2012). Lead toxicity, defense strategies and associated indicative biomarkers in *Talinum triangulare* grown hydroponically. Chemosphere 89, 1056–1065. 10.1016/j.chemosphere.2012.05.070 22722003

[B28] Kupper H. Kupper F. Spiller M. (1996). Environmental relevance of heavy metal substituted chlorophylls using the example of water plants. J. Exp. Bot. 47, 259–266. 10.1093/jxb/47.2.259

[B29] Li J. X. Yang X. E. He Z. L. Jilani G. Sun C. Y. Chen S. M. (2007). Fractionation of lead in paddy soils and its bioavailability to rice plants. Geoderma 141, 174–180. 10.1016/j.geoderma.2007.05.006

[B30] Li M. Ashraf U. Tian H. Mo Z. W. Pan S. Anjum S. A.. (2016). Manganese induced regulations in growth, yield formation, quality characters, rice aroma and enzyme involved in 2-acetyl-1-pyrroline biosynthesis in fragrant rice. Plant Physiol. Biochem. 103, 167–175. 10.1016/j.plaphy.2016.03.009 26995311

[B31] Liu J. Li K. Xu J. Zhang Z. Ma T. Lu X. (2003). Lead toxicity, uptake, and translocation in different rice cultivars. Plant Sci. 165, 793–802. 10.1016/S0168-9452(03)00273-5

[B32] Liu Z. Zhang Q. Han T. Ding Y. Sun J. Wang F.. (2015). Heavy metal pollution in a soil-rice system in the Yangtze river region of China. Int. J. Environ. Res. Public Health 13:63. 10.3390/ijerph13010063 26703698 PMC4730454

[B33] Luna C. M. Gonza'lez C. A. Trippi V. S. (1994). Oxidative damage caused by an excess of copper in oat leaves. Plant Cell Physiol. 35, 11–15.

[B34] Maheshwari R. Dubey R. S. (2009). Nickel-induced oxidative stress and the role of antioxidant defence in rice seedlings. Plant Growth Regul. 59, 37–49. 10.1007/s10725-009-9386-8

[B35] Mishra A. Choudhary M. A. (1998). Amelioration of lead and mercury effects on germination and rice seedling growth by antioxidants. Biol. Plantarum. 41, 469–473. 10.1023/A:1001871015773

[B36] Mishra S. Srivastava S. Tripathi R. Kumar R. Seth C. Gupta D. (2006). Lead detoxification by coontail (*Ceratophyllum demersum* L.) involves induction of phytochelatins and antioxidant system in response to its accumulation. Chemosphere 65, 1027–1039. 10.1016/j.chemosphere.2006.03.033 16682069

[B37] Mittler R. (2002). Oxidative stress, antioxidant and stress tolerance. Trends Plant Sci. 7, 841–851. 10.1016/S1360-1385(02)02312-9 12234732

[B38] Mo Z. Huang J. Xiao D. Ashraf U. Duan M. Pan S.. (2016). Supplementation of 2-Ap, Zn and La improves 2-acetyl-1-pyrroline concentrations in detached aromatic rice panicles *in vitro*. PLoS ONE 11:e0149523. 10.1371/journal.pone.0149523 26910246 PMC4766236

[B39] Mostofa M. G. Hossain M. A. Fujita M. Tran L. S. (2015). Physiological and biochemical mechanisms associated with trehalose-induced copper-stress tolerance in rice. Sci. Rep. 5:11433. 10.1038/srep11433 26073760 PMC4650698

[B40] Piechalak A. Tomaszewska B. Baralkiewicz D. Malecka A. (2002). Accumulation and detoxification of lead ions in legumes. Phytochemistry 60, 153–162. 10.1016/S0031-9422(02)00067-5 12009318

[B41] Piotrowska A. Bajguz A. Godlewska-Zylkiewicz B. Czerpak R. Kaminska M. (2009). Jasmonic acid as modulator of lead toxicity in aquatic plant *Wolffia arrhiza* (Lemnaceae). Environ. Exp. Bot. 66, 507–513. 10.1016/j.envexpbot.2009.03.019

[B42] Pourrut B. Shahid M. Camille D. Peter W. Eric P. (2011). Lead uptake, toxicity, and detoxification in plants. Rev. Environ. Contam. Toxicol. 213, 113–136. 10.1007/978-1-4419-9860-6_4 21541849

[B43] Prasad T. K. Anderson M. D. Martin B. A. Stewart C. R. (1994). Evidence for chilling-induced oxidative stress in maize seedlings and a regulatory role for hydrogen peroxide. Plant Cell 6, 65–74. 10.1105/tpc.6.1.65 12244221 PMC160416

[B44] Qureshi M. Abdin M. Qadir S. Iqbal M. (2007). Lead-induced oxidative stress and metabolic alterations in *Cassia angustifolia* Vahl. Biol. Plantarum. 51, 121–128. 10.1007/s10535-007-0024-x

[B45] Radotic K. Ducic T. Mutavdzic D. (2000). Changes in peroxidase activity and isoenzymes in spruce needles after exposure to different concentrations of cadmium. Environ. Exp. Bot. 44, 105–113. 10.1016/S0098-8472(00)00059-9 10996363

[B46] Reddy A. M. Kumar S. G. Jyothsnakumari G. Thimmanaik S. Sudhakar C. (2005). Lead induced changes in antioxidant metabolism of horsegram (*Macrotyloma uniflorum* (Lam.) Verdc.) and bengalgram (*Cicer arietinum* L.). Chemosphere 60, 97–104. 10.1016/j.chemosphere.2004.11.092 15910908

[B47] Rodriguez C. Santos E. Azevedo R. Moutinho-Pereira J. Correia C. Dias M. C. (2012). Chromium (VI) induces toxicity at different photosynthetic levels in pea. Plant Physiol. Biochem. 53, 94–100. 10.1016/j.plaphy.2012.01.013 22343752

[B48] Rout G. R. Samantaray S. Das P. (2001). Differential lead tolerance of rice and black gram genotypes in hydroponic culture. Rost. Výroba (Praha) 47, 541–548.

[B49] Shahid M. Pinelli E. Pourrut B. Silvestre J. Dumat C. (2011). Lead-induced genotoxicity to *Vicia faba* L. roots in relation with metal cell uptake and initial speciation. Ecotoxicol. Environ. Saf. 74, 78–84. 10.1016/j.ecoenv.2010.08.037 20851467

[B50] Sharma P. Dubey R. S. (2005). Lead toxicity in plants. Braz. J. Plant Physiol. 17, 35–52. 10.1590/S1677-04202005000100004

[B51] Sharma S. S. Dietz K. J. (2009). The relationship between metal toxicity and cellular redox imbalance. Trend Plant Sci. 14, 43–50. 10.1016/j.tplants.2008.10.007 19070530

[B52] Singh R. K. Singh U. S. Khush G. S. (2000). Aromatic Rices. New Delhi: Oxford & IBH Publishing Co. Pvt. Ltd.

[B53] Singh R. Tripathi R. D. Dwivedi S. Kumar A. Trivedi P. K. Chakrabarty D. (2010). Lead bioaccumulation potential of an aquatic macrophyte *Najas indica* are related to antioxidant system. Bioresour. Technol. 101, 3025–3032. 10.1016/j.biortech.2009.12.031 20053550

[B54] Srivastava R. K. Pandey P. Rajpoot R. Rani A. Dubey R. S. (2014). Cadmium and lead interactive effects on oxidative stress and antioxidative responses in rice seedlings. Protoplasma 251, 1047–1065. 10.1007/s00709-014-0614-3 24482190

[B55] Stoltz E. Greger M. (2002). Accumulation properties of As, Cd, Cu, Pb and Zn by four wetland plant species growing on submerged mine tailings. Environ. Exp. Bot. 47, 271–280. 10.1016/S0098-8472(02)00002-3

[B56] Valentovic P. Luxova M. Kolarovic L. Gasparikova O. (2006). Effect of osmotic stress on compatible solutes content, membrane stability and water relations in two maize cultivars. Plant Soil Environ. 52, 186–191.

[B57] Velikova V. Yordanov I. Edreva A. (2000). Oxidative stress and some antioxidant systems in acid rain-treated bean plants: protective role of exogenous polyamines. Plant Sci. 151, 59–66. 10.1016/S0168-9452(99)00197-1

[B58] Verma S. Dubey R. S. (2003). Lead toxicity induces lipid peroxidation and alters the activities of antioxidant enzymes in growing rice plants. Plant Sci. 164, 645–655. 10.1016/S0168-9452(03)00022-0

[B59] Wang X. Wu Y. Y. (1997). Behavior property of heavy metals in soil-rice system. Chin. J. Ecol. 16, 10–14.

[B60] Wang Y. Q. Xiao L. Z. Li S. B. Yang G. Cai X. D. (2010). Effects of combined pollution of Pb and Cd on growth and yield of rice. J. Anhui Agric Sci. 38, 12653–12655. 10.13989/j.cnki.0517-6611.2010.23.214

[B61] Xie Z. M. Huang C. Y. (1994). Relationships between lead zinc arsenic contents and rice tillering in soil-rice system. J. Zhejiang Agric. Univ. 20, 67–71.

[B62] Zhang G. Chen M. Li L. Xu Z. Chen X. Guo J.. (2009). Overexpression of the soybean GmERF3 gene, an AP2/ERF type transcription factor for increased tolerances to salt, drought, and diseases in transgenic tobacco. J. Exp. Bot. 60, 3781–3796. 10.1093/jxb/erp214 19602544 PMC2736888

[B63] Zhang W. F. Zhang F. Raziuddin R. Gong H. J. Yang Z. M. Lu L. (2008). Effects of 5-aminolevulinic acid on oilseed rape seedling growth under herbicide toxicity stress. J. Plant Growth Regul. 27, 159–169. 10.1007/s00344-008-9042-y

[B64] Zhao K. Liu X. M. Xu J. M. Selim H. M. (2010). Heavy metal contaminations in a soil-rice system: Identification of spatial dependence in relation to soil properties of paddy fields. J. Hazard. Mater. 181, 778–787. 10.1016/j.jhazmat.2010.05.081 20561748

[B65] Zhou W. J. Leul M. (1999). Uniconazole-induced tolerance of rape plants to heat stress in relation to changes in hormonal levels, enzyme activities and lipid peroxidation. Plant Growth Regul. 27, 99–104. 10.1023/A:1006165603300

